# The Year and the Century

**Published:** 1901-01-05

**Authors:** 


### THE YEAR AND THE CENTURY.

THE closing of a year which is itself the last year of a century is not only an opportunity for retrospect but an incentive to it, and as the great bell boomed forth the midnight hour, and with impassive monotony hammered out the last stroke of the century, we could not but let our mind drift back, not through the past year alone but through the many which had preceded it, to the time when, a hundred years ago, this wonderfnl nineteenth century was just dawning upon the world. As for the past year we found in its history no cause for exuberant joyfulness. Much as we may wish for peace, and vigorously as we may try to make believe that peace is near, we are still in the midst of war; war is still the main topic, the standing excuse for ill-timed shouts of triumph among the people, as it is for anxiety among those who think; while next to war the most important subject which occupies men's minds is the apparent proof, which has cropped up on many sides, of the extent to which England is being beaten by its many rivals in the perhaps peaceful but none the less vital arena of commerce. Thus the history of the year does not allow of much jubilation to the thoughtful Englishman. Still, when we allowed ourselves to look further back and to consider the whole century rather than the single year, we found some crumbs of comfort in the thought that when it began our forbears were far worse off than we are now; and as we lay listening to the chimes tinkling from many churches on that new year's morn, the bright spot, the one thing that took us from the somewhat dreary prospects of the moment, was the vista of marvellous progress which the history of the century has displayed.

### A HUNDRED YEARS AGO.

In the year 1800 England was still at war with France as she had been for seven long years. Everywhere there was the greatest distress and everywhere the gravest discontent. Income tax was twice as high as it is now, and provisions of every kind were so dear that among all but those who were well off the urgent necessity of obtaining food prevented almost every other kind of expenditure. The farmers and the landowners alone were prosperous, and perhaps for very contrast with the hardships of the poor, the fashion with the wealthy was to gorge themselves with meats and drinks to an extent which in these days we should regard as bestial. Nevertheless, thinkers and investigators were at work even in those dark days. Volta was developing his voltaic pile the essential facts of galvanism were being investigated, machinery was being improved, the steam engine was being turned to great use, experiments in steam navigation were being tried upon the Thames, to be quickly followed by the use of steam on other rivers. In 1801 Parliament sanctioned the construction of the first iron railway in England, which ran from the Thames, at Wandsworth to Croydon, horses being the motive power. On January 13th, 1800, the Royal Institution of Great Britain, which had been founded the year before, was incorporated by Royal Charter, and in many directions signs were not wanting that peace was the one thing lacking to render possible the great prosperity that was to follow.

### SANITARY EXPENDITURE.

The great drain upon the resources of the country, entailed by the prolonged wars in which all Europe had been involved, rendered it impossible to undertake that great expenditure for local and sanitary improvements which has been so marked a feature of municipal life during the last half, and especially the last quarter, of the century, an expenditure in return for which we have no doubt received good value, but which, for all that, has involved almost every district in such an amount of local debt as to cause much anxiety for the future. Accustomed as we are to the free use of the hundred and one comforts and conveniences provided by that *deus ex machina* "the rates," it is difficult for us to realise how little was done for the town dweller in former times to render his life easy.

When we consider the dim, ill-lighted streets, with an occasional oil lamp swung across the principal thoroughfares, the wretched pavements, the lack of drains, the miserable water supply, the absence of all that assistance in difficulty and protection in danger which we associate with the word "policeman;" and when we further remember that easy as locomotion might be for the rich, there were no cabs and none of those omnibuses and tramcars which now turn the streets of London into a "moving platform" open for the use of anyone who has a penny in his pocket, and that our modern postal and telegraph and telephone facilities were utterly undreamed of, we can understand how very different was then not only the mode of life but the prevailing character of the people.

### THE NATIONAL CHARACTER.

Truly every man's house was then his castle, as we pretend it is now. But a man then had to defend himself and his house and his family; he had to provide for himself or starve; it was a case of "sink or swim," and all depended on the individual. If a man chose to have a family of children he had to educate them himself and at his own cost, and bring them up to some trade or business, or have them thrown upon his hands. There was less orderly obedience to law in those days than there is now; but at the same time there was less of that helpless dependence upon the authorities in every little domestic circumstance which is so characteristic of the present time. If their children had a rash they did not send them to hospital at the public expense; if the kitchen chimney got on fire they did not find a fire-engine galloping round the corner in a couple of minutes; if a stranger knocked over loudly at the gate they did not whistle for a policeman. They had to meet the difficulty as best they could. And all this made for character.

### PATHOLOGY.

Turning now to the condition of the medical sciences a hundred years ago, it is interesting to note that although physicians, surgeons, and apothecaries were all busily at work treating disease, and treating it very vigorously, too, they were all alike absolutely without any knowledge of pathology in the sense in which that word is commonly used nowadays. Macroscopical morbid anatomy of course they had, but seeing that they had no knowledge of microscopical anatomy, no knowledge of pathological chemistry, and only a fantastic knowledge of any chemistry at all, and that parasitology, except so far as a few worms and hydatids were concerned, was an absolute blank, we need hardly be surprised that as to the processes of disease they knew nothing. At the same time it is to be admitted with all humility that although the century has provided us with a multitude of observations, and although we know

B

infinitely more than they did a hundred years ago about the departures from the normal which are met with in disease, much that goes for knowledge as to the processes by which these things are brought about is even now largely a matter of conjecture and more or less plausible hypothesis. Facts the century has given us in plenty; but the light that shall lead on to the secrets of life, and thus of disease, is still hidden from us.

The Humanity of Modern Medicine.

Among the changes in the practice of our profession during the century, one of the most striking is its increased humanity. We consider the feelings of our patients as well as their diseases, we seek to comfort if we cannot cure, and, above all things, we endeavour not to hurt. Of this no better example could be given than the vast change which has come over the treatment of the most helpless of all patients—those who are afflicted in mind. If we think of the chains and the manacles which were used for the restraint of lunatics a hundred years ago, the iron collars by which they were fastened to the walls, the iron frames in which they were confined, the muzzles, the girdles, the handcuffs, and the leg-irons which were then thought necessary for the treatment of lunatics, and, worse than all, of the free use of corporal chastisement which was then employed, not for the maintenance of discipline, but as a part of the mode of cure, and if we compare, with the condition of affairs which these things display, the palaces which are now provided for these unfortunate people and the enormous expense to which the country voluntarily puts itself lest, perchance, a lunatic here and there should end his useless life, we see how great a difference a hundred years has made in the way in which such matters are regarded. The same feeling of tenderness towards the patient which has so immensely improved the lot of the lunatic has extended in every direction, as shown in the use of anæsthetics in every department even of minor surgery, and in the keen competition which has taken place with the object of rendering even the giving of anæsthetics as little unpleasant as possible.

But in every direction the same thing is seen. Whether by the provision of skilled nurses, by the abolition of filthy medicines, by the sugar coating of pills, by the giving of anæsthetics, or even by the bland and gentle " bed-side manner" which is thought to lead to practice we find on every hand as one of the characteristics by which the medicine of to-day differs from that of the gruff period at the beginning of the century an ever-present humanity, and a constant desire to lessen even the smaller sufferings of our patients. As for surgery, the difference between the beginning and the end of the century is absolutely indescribable, the introduction of anæsthetics and of antiseptics having produced a complete revolution. This, however, has now become a tale almost too often told.

The Progress of the Year.

Except in regard to the clinching proof, given by actual experiment of the truth of the mosquito theory regarding the propagation of malaria, it must be confessed that no very great strides have been made during the year in the art and science of medicine. There have been, however, a considerable number of notable events of considerable interest to medical men.

The Plague.

The invasion of these islands by a definite outbreak of plague had long been anticipated by those who had watched the progress of that serious disease, and this year it came. How it came, by what route it arrived, nay, who was the first person to be attacked by it, we do not, and probably never shall know, but in August last, after an absence from our shores of more than two hundred years, plague broke out in Glasgow. The epidemic, if so it could be called, was quickly brought under control. In other words it ceased. But although the cases were not many, the fatality among those which were definitely diagnosed as plague was considerable, thus affording another illustration of the fact that although plague may not easily be imported into these latitudes, its virulence, when once it becomes established, is by no means to be made light of. The methods adopted in Glasgow were those rendered available by the general sanitary machinery of the country, namely, isolation in hospital of all cases, both established and suspected, house-to-house visitation in search of cases presenting suspicious symptoms, bacteriological examination in doubtful cases, thorough cleansing of the districts in which the disease had occurred, and isolation for a time followed by more prolonged observation of those who were known to have been exposed to infection.

The Powers of Our Sanitary Authorities.

The fact that so highly diffusible an infection as that of plague should have so speedily come to nothing is not only a matter for sincere congratulation, but may perhaps be taken as a lesson, for it shows what sanitary measures can do if really put in force with vigour against a zymotic disease. The sanitary authorities have many powers, some of which they exercise in a more or less methodical and routine manner. As a rule, however, we take things easily and accept with perhaps more than due indifference the fevers, the measles, the whooping coughs, the diarrhœas, by which we, and especially the younger members of the community, are decimated year by year. We have not yet got out of the mediæval fashion of regarding these things as the gifts of providence, a providence against which to kick is vain. Plague, however, is another affair, it is not within our category of essentials, we resist it might and main, and it fades away. And so perhaps might other more fully domesticated maladies were they resisted in the same hearty fashion. And this is the lesson. As a matter of fact cholera and plague seem to be the only two diseases against which our sanitary organisation has hitherto thought it worth while to put forth its full strength, and in both cases the results have been eminently satisfactory. Perhaps other diseases might prove equally complaisant if we did but grasp the nettle with sufficient firmness.

Enteric Fever.

By contraries this brings us to the question of enteric fever, a disease which has shown itself peculiarly masterful in South Africa, and in regard to which endless discussions have taken place throughout the year. It cannot be said that either the experience or the discussions have added much to our knowledge. We knew pretty well before the war how typhoid fever was spread, and the terrible outbreak which has so hampered the conduct of the war has but proved that our knowledge on the subject was right. War is war, however, and we are told that one of the risks which troops must always run is that of being decimated by typhoid fever, that it is all in the day's work whether they are hit by a bullet or a microbe. Still we do not think that the last word has been said in regard to prevention. Undoubtedly the teaching of the campaign so far as typhoid is concerned is that in the conduct of a war both officers and men must learn not merely to put up with but to actively assist in intelligently carrying out a sanitary discipline far stricter than anything that was at all attempted in South Africa until after typhoid had obtained a footing among our troops. In fact we may doubt whether, even after all the lessons that have been given us, the sanitary organisation of our camps is yet such as would satisfy the medical officers of health of some of our great towns.

### Bullet Wounds.

The surgical observations of the year have been concerned chiefly with bullet wounds, with operations on the stomach and the pancreas, and with the use of massage and more or less immediate movement in the treatment of certain kinds of fracture. There can be little doubt that many of the crude theories which were put forward at the time of the Greco-Turkish War in regard to the action of high velocity bullets will have to be modified. The fact is being appreciated that the problem of the action of a bullet impinging upon the human body is a very much more complex one than some people have appeared to think, and we have no doubt that when the experience of this war comes to be carefully collated a great deal of useful information on the subject will be elicited. In the meantime the great lesson has been driven home that no pains can be too great to take to ensure the highest degree of asepticity which can be attained under the trying circumstances of war.

### The Great Desideratum.

The great desideratum still seems to be some means of securing asepsis without the use of water. So free is the use of water in civil hospitals, and so dependent have we all become on washing as a way of getting clean, that when surgeons trained in hospitals and accustomed to hospital surroundings find themselves confronted with the grimy facts of war, and are forced to treat as best they may crowds of blood-stained men, soaked in dirt, and clad in filthy clothes that may not have been changed for weeks, they are apt to be seized with a feeling of helplessness in view of the impossibility of bringing into play any of the antiseptic methods they have been accustomed to. The impossibility of obtaining water for surgical purposes is one of the striking experiences of this war. But those who look back know that exactly the same thing has been felt over and over again—notably so recently as in the Chitral Expedition. Some steps have been made in the past year in the direction of working out a non-water-using antiseptic method, and in this direction we must look for much advance. Unless the water is far purer than much that could be obtained at many an urgent moment, its use, even for cleansing purposes, is full of risk. In fact, from a surgical point of view there can be no doubt that the patient's blood was far the cleanest and most aseptic thing on many a field dressing station. Under such circumstances a new and entirely special system of antiseptic routine becomes essential for use in war.

### Spinal Cocainisation.

A very striking innovation has been brought prominently before the profession during the past year, although the experiments on which it is founded are of earlier date. This is the production of anæsthesia by the injection of cocaine within the spinal membranes. To this we have several times referred, as one observer after another has reported on the subject. That it can be done, and that the lower segment, at any rate, of the body can be so deprived of sensation as to render it quite possible to perform an operation while the patient looks on, retaining his full consciousness, is clear enough. But the performance has risks of its own which seem inseparable from it, while the risks which would be run were the injection carelessly performed without the most complete precautions for the maintenance of asepsis, would be extreme. The experiments are going on apace, and some definite conclusion ought to be arrived at before long. The steady extension of the practice of obtaining local anæsthesia by infiltration of the tissues with eucaine is another affair. Considerable experience in this method has been gained during the year, and one can hardly doubt that its use in certain cases in which general anæsthesia is attended with much peril it will be found very useful, offering, as it will, the chance of operative help to many cases which otherwise might be hopeless.

### The Disappointing End of a Brilliant Century.

Surveying the whole field of medical progress it cannot be said that in any department the year 1900 has marked itself out from other years as one of great inventions and striking advance. Progress there has been, of course. Men cannot work as medical men are always working without adding every year, even considerably, to the sum of medical knowledge; nor can great congresses, such as have drawn representatives of all nations to Paris during the time of the International Exposition, take place without an amount of interchange of thought which, although not itself productive, sows the seed for further advances, but of great medical inventions the closing year of the nineteenth century is sadly barren, and to Englishmen it is to be feared that, so far as its medical history is concerned, the year will be remembered for nothing so much as for a great catastrophe and a great disappointment—that we, a people who pride ourselves upon being pioneers in sanitary progress, should have allowed our army to be ravaged by fever, and should very nearly have allowed the fruits of our hard-won victories to be snatched from us, and all at the hands of that very disease our triumph over which in civil life is one of the constant boasts of English sanitarians. To us, to all Englishmen who believe in science, and in the life-saving utility of science if its teachings are fully accepted and followed without fail, this has been a sad ending to a brilliant century. Let us hope that we shall do better in the one into which we are now entering. We are taught from our childhood that "knowledge is power." But knowledge is not power unless it lead to action, and science is but of little benefit to nations unless those in authority will loyally and even humbly accept its teachings.

### Homage to the Dead.

Finally we must do homage to the great men who during the past year have been claimed by death. Sir Thomas Grainger Stewart, Sir Henry Acland, Sir William Priestley, Dr. Dennis Embleton—one of the oldest members of the profession, Professor Alfred William Hughes, Sir William Stokes, William Anderson, F.R.C.S., John Neville Colley Davies-Colley, F.R.C.S., George Viner Ellis, F.R.C.S., Thomas Jones, F.R.C.S., Daniel John Leech, F.R.C.S., Lewis Albert Sayre, M.D., are but a few out of the long list of those who have worked hard and well for the advancement of knowledge and the good of humanity, and after gaining honour and high repute have now gained rest.

